# 50 Hz Electromagnetic Field Produced Changes in FTIR Spectroscopy Associated with Mitochondrial Transmembrane Potential Reduction in Neuronal-Like SH-SY5Y Cells

**DOI:** 10.1155/2013/414393

**Published:** 2013-07-16

**Authors:** Emanuele Calabrò, Salvatore Condello, Monica Currò, Nadia Ferlazzo, Mercurio Vecchio, Daniela Caccamo, Salvatore Magazù, Riccardo Ientile

**Affiliations:** ^1^Department of Physics, University of Messina, 98165 Messina, Italy; ^2^Department of Biomedical Sciences and Morphofunctional Imaging, University of Messina, 98125 Messina, Italy

## Abstract

SH-SY5Y neuroblastoma cells were used as an experimental model to study the effects of 50 Hz electromagnetic field, in the range from 50 **µ**T to 1.4 mT. Fourier transform infrared spectroscopy analysis evidenced a reduction in intensity of the amide A band and a slight increase of vibration bands at 2921 cm^−1^ and 2853 cm^−1^ corresponding to methylene groups. A further increase of the magnetic field intensity of exposure up to 0.8 mT and 1.4 mT produced a clear increase in intensity of CH_2_ vibration bands. Moreover, it has been observed some alterations in the amide I region, such as a shifted peak of the amide I band to a smaller wavenumber, probably due to protein conformational changes. These results suggested that exposure to extremely low electromagnetic fields influenced lipid components of cellular membrane and the N–H in-plane bending and C–N stretching vibrations of peptide linkages, modifying the secondary structures of **α**-helix and **β**-sheet contents and producing unfolding process in cell membrane proteins. The observed changes after exposure to 50 Hz electromagnetic field higher than 0.8 mT were associated with a significant reduction of cell viability and reduced mitochondrial transmembrane potential.

## 1. Introduction

Although 50/60 Hz EMF seems to not directly lead to genotoxic effects, it is possible that certain cellular processes altered by exposure to ELF-EMFs indirectly affect the structure of DNA, causing strand breaks and other chromosomal aberrations [1].

Several epidemiological studies reported a relationship between an increase of risk of cancer and the exposure to ELF-EMF. In particular three studies of the World Health Organization (WHO) on EMF evidenced possible health effects from exposure to static and ELF-EMFs [2–4].

Environmental Health Criteria (EHC) delineated the main objectives to review the scientific literature on the biological effects of exposure to ELF-EMFs to use this health risk assessment to make recommendations to national authorities on health protection programs.

EMFs are generated everywhere in our living environment by modern electrical systems such as power lines, electric generators and motors, electrical wiring, home electronic devices, and wireless communication systems [5].

The electric field inside the body is normally five to six orders of magnitude smaller than the external electric field, whereas the permeability of tissue relative to magnetic fields is the same as that of air, so that the field in organic system is the same as the external field. From this evidence many investigations emphasized the effects of magnetic field component with respect to those due to electric component effects.

In the close proximity of certain home appliances, the magnetic-field intensities can be as much as few hundred microteslas, whereas in some workplaces they can reach 10 mT, leading us to investigate the range of exposure to magnetic field around a few milliteslas.

The exposure of organic systems to ELF-EMF can interfere with some metabolic processes, modifying intracellular enzymatic pathways and causing an increase in the production of free radicals which may alter or interfere with DNA reparation or replication mechanisms and protein and lipid-containing structures [1].

The redox status can be abled to regulate gene expression determining changes in cell growth, differentiation, proliferation, and stress response. 

Thus, EMF might be a stimulus to induce an activated state of the cell such as phagocytosis, which then enhances the release of free radicals, in turn leading to genotoxic events. EMF exposure can cause both acute and chronic effects that are mediated by increased free radical levels.

An increase in the lifetime of free radicals by EMF leads to persistently elevated free radical concentrations. In general, reactions in which radicals are involved become more frequent, increasing the possibility of DNA damage.

Several studies have shown no genotoxic effects after exposure to ELF-EMFs in several types of mammalian cells [6–9].

In contrast, other studies demonstrated genotoxic damage in various cell models [10–12]. In this regard, lymphocytes first exposed to the ELF field for 24 h at 80 or 800 *μ*T showed significant increases in the frequency of micronuclei and apoptosis after incubation with different doses of vinblastine [13]. Other authors investigated the effect of ELF-EMF on the expression of heat shock proteins (HSPs). Hsp proteins are inducible after the cells have been exposed to a wide range of stress signals and have also been shown to be expressed at atypical levels in tumour cells or tissue. Such observations have led to suggestions that HSPs could be used as biomarkers for cellular stress in general. 

The transcription of the heat shock genes hsp70 was shown to increase because of the exposure to a 60 Hz, 8 *μ*T magnetic field [14]. Pipkin et al. [15] showed that inducible hsp70 (hsp70B) was overexpressed after 60 Hz ELF magnetic field exposure at 1 mT.

In neuroblastoma cells, no effects were found in nerve cells, but a decrease in the conductance of gap junction channels under exposure to 20 mA m-2 at 60 Hz and a significant increase in intracellular Ca^2+^ at current densities of more than 10 mA m-2 were found [16].

According to previous observations, in the present study the parameters of time exposures and EMF intensities were 5–24 hours and 50 *μ*T–1 mT, respectively. 

This study was aimed to investigate whether the exposure to electromagnetic fields at the frequency of 50 Hz may be considered as an environmental insult by examining mitochondrial transmembrane potential in SH-SY5Y neuroblastoma cells, differentiated to dopaminergic neuron-like cells. Further FTIR spectroscopy was used to study the relationship between conformational changes of protein and DNA structures and cell damage. 

## 2. Materials and Methods

The human neuroblastoma cell line SH-SY5Y (CRL-2266) was purchased from the American Type Culture Collections (ATCC) (Rockville, MD, US). Fetal bovine serum (FBS), antibiotics, minimum essential medium (MEM) eagle (M5650), Nutrient Mixture F-12 Ham (M4888), all-trans retinoic acid (RA), sodium pyruvate, phosphate buffered saline (PBS) solution, and other chemicals of analytical grade were from Sigma, Milan, Italy. 

### 2.1. Cell Culture and Treatment

Human neuroblastoma cell line SH-SY5Y cells were cultured in a 1 : 1 mixture of MEM and Ham's F-12 medium containing 10% (v/v) heat inactivated FBS, L-glutamine (2 mM), and sodium pyruvate (1 mM) and maintained at 37°C in a humidified incubator with 5% CO_2_ and 95% air.

Subconfluent cells were washed twice with PBS then incubated in MEM/Ham's F-12 medium containing 10 *μ*M RA (10 mM in dimethyl sulphoxide (DMSO) stock solution), 1% FBS, L-glutamine (2 mM), and sodium pyruvate (1 mM). The medium was renewed every two days.

After 5 days of 10 *μ*M RA exposure, differentiated SH-SY5Y cells were exposed to magnetic field.

### 2.2. Experimental Design

The exposure system consisted of a couple of Helmholtz coils, with pole pieces of round parallel polar faces, to produce a uniform magnetic field at the center of the coils distance.

This device was used to generate time-varying electromagnetic fields at the frequency of 50 Hz by means of a AC voltage, which enabled us to change the magnetic flux density between the polar faces of the coils. RA differentiated SH-SY5Y cells, grown in both 25 cm^2^ culture flasks or in 96-well plates, were exposed to magnetic field. Samples were placed at the centre of a uniform field area between the coils.

The coils aligned on a common axis were wound in the same sense and connected in series.

The value of the coil spacing was assumed to be equal to the coils radius *R* = 150 mm, because this experimental setup showed that the coils fields add in such a way that there is a region around the geometric centre where the magnetic field has constant magnitude and angle.

This experimental setup provided that the magnitude of the magnetic field is linearly proportional to the applied current through the two coils and it is given by *B* = (4/5)^(3/2)^(*μ*_*o*_ *NI*)/*R*, where *N* is the number of turns per coil (*N* = 124), *μ*_*o*_ = 4π*10^−7^T · m/A is the magnetic permeability, and *I* is the coil current.

However, the uniformity of the magnetic field intensity was continuously monitored by the magnetic field probe GM07 Gaussmeter (HIRST-Magnetic Instruments Ltd, Falmouth, Cornwall, UK) within a range of 2 cm around the centre of the coils distance, where samples of human SH-SY5Y neuroblastoma cells were placed.

The coils were located into a incubator in a 5% CO_2_/95% air humidified at the temperature of 37.1°C (incubator series 5400-115 V models, Thermo Electron Corporation, Winchester, VA, USA).

Not exposed samples were placed into another incubator of the same model, at the same physical conditions, maintained rigorously at the values of air humidified incubator and temperature previously reported. Preliminary experiments for thermal simulation were performed. Inside the culture medium, temperatures are monitored with accurate Pt100 probes, using hand-held thermometer model CTH 6200 (from Wika Wiegand GmbH & Co., Klingenberg, Germany). During the exposure, no significant increase in the temperature (±0.1 T°C) was observed. 

### 2.3. Infrared Spectroscopy

FTIR spectra of SH-SY5Y neuroblastoma cells, exposed and not exposed, were recorded at room temperature by a spectrometer Vertex 80 v from Bruker Optics.

For FTIR analysis cellular cultures were disaggregated from the culture medium using a trypsin solution to form single-cell suspensions [17, 18] and were placed upon CaF_2_ windows for FTIR measurements.

The attenuated total reflection (ATR) method was chosen for spectrum collection.

In fact, ATR for cells spectra collection in FTIR was improved, because it was no dependent on sample thickness [19].

Furthermore, ATR technique is the desired method to overcome solvent masking since the penetration depth of infrared light is inherently limited to a fraction of the wavelength estimated to be *λ*/10, permitting rapid secondary structure analysis on small volumes [20].

For each spectrum, 128 interferograms were collected and coadded by Fourier transformed employing a Happ-Genzel apodization function to generate a spectrum with a spectral resolution of 4 cm^−1^ in the range from 5000 cm^−1^ to 1000 cm^−1^.

IR spectra of water solution were subtracted from acquired spectra at the corresponding temperature. Each measure was performed under vacuum to eliminate minor spectral contributions due to residual water vapor, and a smoothing correction for atmospheric water background was performed.

The IR spectra were baseline-corrected by means of automatic baseline scattering correction function, to subtract baselines from spectra, which allows getting spectra with band edges of up to the theoretical baseline.

The spectra were successively area-normalized for exposed cells and control samples, and vector normalization was used, calculating the average value of the spectrum and subtracting from the spectrum decreasing the mid spectrum. The sum of the squares of all values was calculated, and the spectrum was divided by the square root of this sum.

Interactive baseline rubberband correction was used to minimize the water band contribution to spectra. This method also uses a rubber band which is stretched from one spectrum end to the other, and the band is pressed onto the spectrum from the bottom up with varying intensity. This method performs iteratively, depending on the number of iterations in the algorithm and the baseline as a frequency polygon consisting of *n* baseline points. The resulting spectrum will be the original spectrum minus the baselines points manually set and a subsequent concave rubberband correction. We used the default value of *n* = 64 baseline points and a number of 60 iterations.

ATR spectra were smoothed by the Loess algorithm, and the deconvolved spectra were fitted with Gaussian band profiles. 

Second-derivative analysis of infrared spectra was performed to enhance spectral features [21, 22].

Both exposed and control samples were located in the same room at a temperature of 20°C.

### 2.4. Evaluation of Cell Viability

Cell viability was evaluated by an MTT quantitative colorimetric assay. After exposure to magnetic field, RA differentiated SH-SY5Y cells, grown in 96-well culture plates at a density of 5 × 10^4^ cell/well, were incubated with fresh medium containing MTT (0.5 mg/mL) at 37°C for 4 h. Then, insoluble formazan crystals were dissolved in 100 *μ*L of a 10% (w/v) sodium dodecyl sulfate solution in HCl 0.01 M for 10 min. The optical density in each well was evaluated by spectrophotometrical measurement. Absorbance was determined at 570 nm using a microplate reader (Tecan Italia, Cologno Monzese, Italy).

### 2.5. Measurement of Mitochondrial Transmembrane Potential (Δ*ψ*_*m*_)

Alterations in mitochondrial transmembrane potential (Δ*ψ*m) were assayed by the incorporation of a cationic fluorescent dye rhodamine 123. After treatments as previously described, the cells (2.5 · 10^5^ cells/mL in 6-well plates) were changed to fresh medium containing 10 *μ*M rhodamine 123 and incubated for 15 min at 37°C.

The cells were then collected and washed twice with PBS (pH 7.4), and the fluorescence intensity was analyzed at wavelength of 488 nm excitation and 525 nm emission under fluorescein optics.

### 2.6. Statistical Analysis

All values are expressed as mean ± standard error of the mean (SEM). Statistical analysis was carried out using Student's *t*-test for comparisons between two groups, with *P* values less than 0.05 considered significant.

## 3. Results and Discussion

The range of exposure of the magnetic flux density was chosen from 50 *μ*T to 1.4 mT, following values of exposure used in previous research, as reported in the first section of this paper. Otherwise, the values selected for this study are around the reference, based on the guidelines of the International Commission of Non-Ionizing Radiation Protection [23].

FTIR techniques can easily investigate the spectral region covering the range between 14000 and 20 cm^−1^, but only in the midinfrared region, 200 to 4000 cm^−1^ is used for analysis of biological materials [24], where absorption spectra of the compounds are characterized by the functional group frequencies of the molecules, which are sensitive to any environmental changes or the changes in their structures and conformations.

Previous FTIR spectroscopic analysis of dying cells has shown that two main characteristic spectral signatures can be assumed as indicative of death [18, 25].

In particular, we observed the shift down of the protein amide I and amide II peak's centroid, indicating a change in the overall proteins conformational states within the cell and an increase in the vibration band at 1740 cm^−1^.

**Figure 1:**
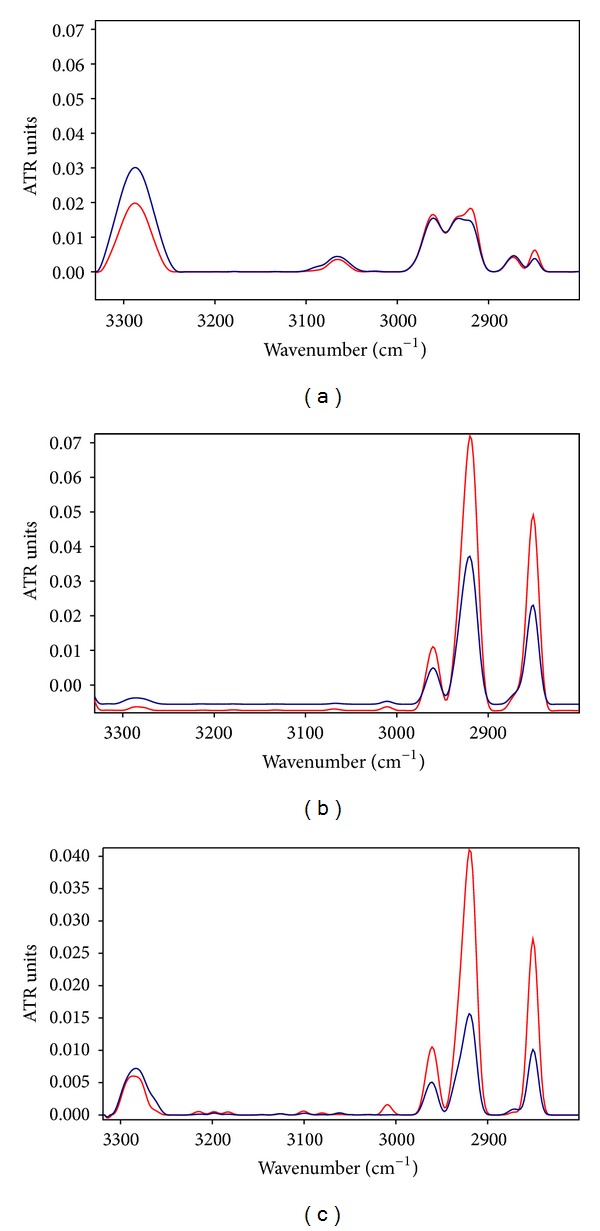
ATR spectra of neuroblastoma cells in the spectral IR region from 3300 to 2800 cm^−1^: (a) after 4 h of exposure to 50 Hz EMF at 50 *μ*T; (b) after 4 h of exposure to 50 Hz EMF at 0.8 mT; (c) after 4 h of exposure to 50 Hz EMF at 1.4 mT. The increase in intensity of CH_2_ asymmetric and symmetric stretching appeared clearly after exposure to the magnetic flux densities of 0.8 mT and 1.4 mT.

**Figure 2:**
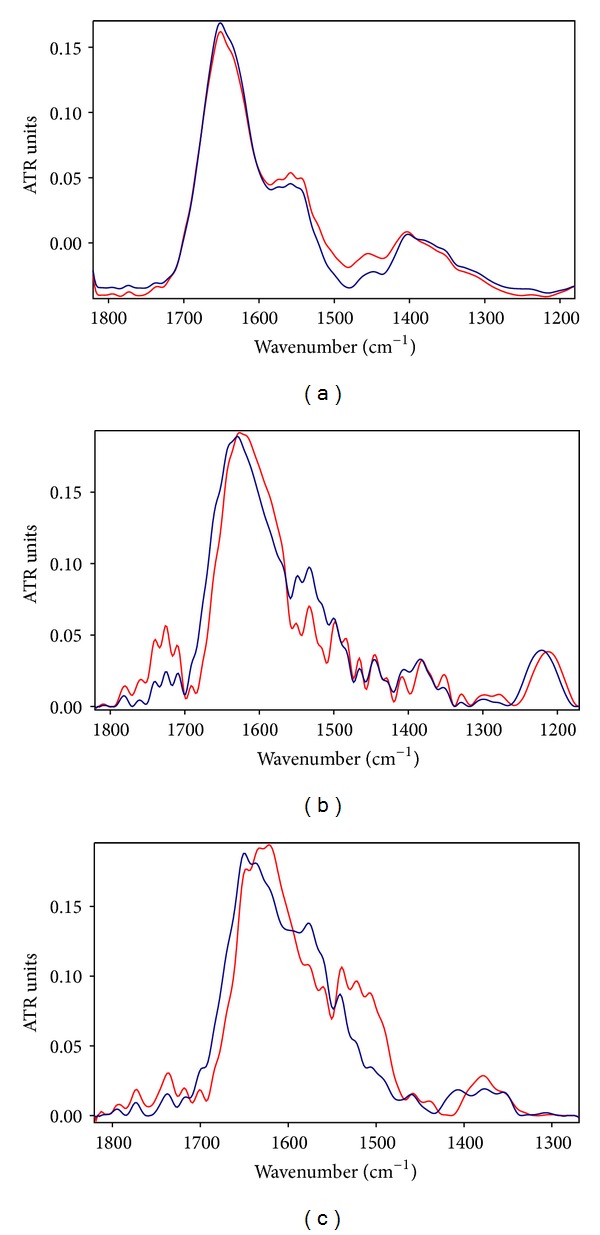
ATR spectra of neuroblastoma cells in the spectral IR region from 1800 to 1250 cm^−1^. (a) after 4 h of exposure to 50 Hz EMF at 50 *μ*T; (b) after 4 h of exposure to 50 Hz EMF at 0.8 mT; (c) after 4 h of exposure to 50 Hz EMF at 1.4 mT. The increase in *β*-sheet content with respect to *α*-helix component in amide I region and the increase at 1740 cm^−1^ vibration band in the amide I region appeared clearly after the exposure to 50 Hz EMF at the intensity of 0.8 mT. The *β*-sheet content increased more after exposure to 1.4 mT, as reported in Table 2.

The exposure to ELF-EMF at the low intensity of 50 *μ*T for 4 h produced a decrease in the amide A intensity vibration band around 2995 cm^−1^ (Figure 1(a)), whereas no changes was produced in amide I and amide II bands (Figure 2(a)).

The amide A band, due to the peptide linkage N–H stretching mode, can be considered a useful marker of secondary structure, which often appears as a doublet band arising from two different structures, the stronger of the two components being associated with the standard *α*-helical structure in the chain [26, 27].

Previous research showed that local environments and hydrogen bonding configurations can play a role in determining the line shape of amide A vibration [28–30].

A decrease of amide A after exposure to ELF-EMF was already observed in FTIR spectra of haemoglobin aqueous solution after 3 hours of exposure to 50 Hz frequency EMF at 1 mT [31].

In addition, a low increase of the vibration bands at 2921 and 2853 cm^−1^ occurred after exposure to 50 Hz EMF at 50 *μ*T, (Figure 1(a)).

Further exposures of 4 h at the magnetic flux densities of 0.8 mT and 1.4 mT were carried out, whose representative spectra in the same region 3300–2800 cm^−1^ are represented in Figures 1(b) and 1(c), respectively, showing a relevant increase in intensity of CH_2_ group at 2921 and 2853 cm^−1^ of exposed samples.

The band near 2853 cm^−1^ is due to the symmetric ^s^CH_2_ stretching of the methylene chains in membrane lipids or proteins; the peak around 2925 cm^−1^ is due to the asymmetric ^as^CH_2_ stretching [32, 33]. Otherwise, the bands at 2961 and 2871 cm^−1^ can originate, respectively, from the asymmetric ^as^CH_3_ and the symmetric stretching vibrations ^s^CH_3_ of CH_3_ methyl groups of lipids or protein side chains [34, 35].

In order that CH_2_/CH_3_ ratio was quantified, the ratio between the integrated area of 2925 cm^−1^ ^as^CH_2_ band (computed from 2945 to 2910 cm^−1^) and the integrated area of 2961 cm^−1^ ^as^CH_3_ (computed from 2980 to 2945 cm^−1^) of exposed samples and that relative to not exposed samples were calculated.

Analogue calculation was carried out as to the ratio between the integrated area of 2853 cm^−1^ ^s^CH_2_ band (evaluated from 2835 to 2875 cm^−1^) and the same integrated area of 2961 cm^−1^ ^as^CH_3_.

These computations have been summarized in Table 1.

Statistical analysis established significant difference in comparison to controls for the CH_2_/CH_3_ changes after the exposures to 50 Hz EMF at the intensity of 0.8 mT, (*P* < 0.001).

However, the change observed in the CH_2_/CH_3_ ratio must not be necessarily associated to apoptosis, as it could be produced by cell growth, or a decrease in cellular volume to which would correspond an increase in the surface area. This may enrich the overall cellular content with fatty acids and phospholipids and a relative increase of CH_2_ as to CH_3_ groups.

Exposure to ELF-EMF at 0.8 mT produced evident changes in amide I and amide II regions as represented in Figure 2(b) in which these regions were zoomed in, whereas no appreciable changes occurred in these regions after exposure at the low intensity of 50 *μ*T (see Figure 2(a)).

The peaks observed around 1650 cm^−1^, 1635 cm^−1^, 1675 cm^−1^, and 1640 cm^−1^ in the amide I region can be assigned to *α*-helices, *β*-sheets, turns, and random coil, respectively [36].

The analysis of exposed spectra at the intensity of 0.8 mT revealed a loss of *α*-helical and short segment connecting *α*-helix segments content and an increase of the *β*-sheet component at 1635 cm^−1^ relative to the *α*-helix, as reported in Table 1.

The *β*-sheet/*α*-helix ratio of the integrated areas of their vibration bands for the exposed samples was higher in comparison to controls confirming a significant increase of the *β*-sheet content (1.29 ± 0.05) with respect to the *α*-helix (1.13 ± 0.04) (*P* < 0.05) in the proteins secondary structure.

These results indicated changes in the overall protein conformational state within the cell, which could be due to denaturation of protein during apoptosis or, alternatively, to different distributions of proteins and unfolding process and formation of aggregates [37].

In the amide II region the absorption band around 1545 cm^−1^ can be assigned to *α*-helix structure, and vibration bands close to 1525 and 1535 cm^−1^ may be attributed to *β*-sheet and random coil, respectively [22].

However, the relative increase of *β*-sheet content as to the *α*-helix component in this region after exposure was lesser evident than that occurred in amide I region.

The peak at 1740 cm^−1^ assigned to nonhydrogen-bonded ester carbonyl C=O stretching mode within phospholipids increased heavier after the exposure, as can be observed in Figure 2(b), giving further evidence of a dying cell state as suggested by [38, 39]. The integrated area of 1740 cm^−1^ band (computed from 1725 to 1760 cm^−1^) of exposed samples and that relative to not exposed samples were computed and reported in Table 1, providing that the 1740 cm^−1^ band increased significantly with respect to the band at 1725 cm^−1^ (2.03 ± 0.15 versus 1.25 ± 0.10) after the exposure (*P* < 0.005).

This result suggest that the C=O ester carbonyl groups of lipids in the cell are becoming predominantly nonhydrogen bonded, which would be in agreement with occurring oxidative damage. Indeed, apoptosis should be associated with increased oxidative damage [40, 41].

The influence of exposure to ELF-EMF on the DNA of treated cells can be observed from IR bands due to vibrations of various structural groups in DNA such as the two phosphate absorption bands around 1235 cm^−1^ and 1080 cm^−1^, that correspond to the asymmetric stretching phosphate mode ^as^PO_2_^−^ and symmetric stretching phosphate mode of phosphodiester bonds ^s^PO_2_^−^ in nucleic acids, respectively [32, 33]. 

The integrated area of ^as^PO_2_^−^ and ^s^PO_2_^−^ of exposed samples in comparison to control samples, computed from 1260 to 1200 cm^−1^ and from 1120 to 1020 cm^−1^, respectively, decreased significantly (*P* < 0.05) after the exposure to ELF-EMF at 0.8 mT, as reported in Table 1.

In addition, the peak's centroid of   ^as^PO_2_^−^ and  ^s^PO_2_ bands shifted 9 cm^−1^ and 3 cm^−1^, respectively after exposure, as can be observed in Figure 2(b).

Dependent on EMF field intensity, we observed a significant reduction of the levels of the bands suggesting a decrease in DNA content in the cells exposed to ELF-EMF.

Further exposure of 4 h to 50 Hz EMF at the magnetic flux density of 1.4 mT produced heavier significant increases of CH_2_/CH_3_ ratio (*P* < 0.001), as shown in Figure 1(c) and reported in Table 1.

In addition, the *β*-sheet/*α*-helix ratio in amide I and the 1740 cm^−1^/1725 cm^−1^ ratio increased getting to the values of 1.95 ± 0.10 versus 1.31 ± 0.08 and 2.88 ± 0.07 versus 1.14 ± 0.04, respectively, as represented in Figure 2(c) and reported in Table 1, providing further evidence for the dying cells state, which was confirmed by MTT analysis (Table 2).

As reported in Table 2, the 4 h exposure to ELF-EMF (1.4 mT) produced a reduction by 15% of cell viability (*P* < 0.01). According to these results, significant decreases in cells viability of neuronal-like were already observed after exposures of 2 h and 4 h to EMF at 1800 MHz [42].

Furthermore, after 4 h exposure to 50 Hz EMF at 1.4 mT heavy reduction (53%) of mitochondrial transmembrane potential was observed (Table 2). The results confirm several observations suggesting that mitochondria can be the source of energy as well as the source of signals that initiate apoptotic cell death.

A significant loss of ΔΨm renders cells depleted of energy; given the energy needs of neurons, defects in mitochondrial dynamics lead to neuronal cell death.

## 4. Conclusions

To summarize, the exposure to 50 Hz electromagnetic field higher than 0.8 mT was able to produce conformational changes in different biological structures as evidenced by FITR spectroscopy, and these alterations can be associated to reduction of mitochondrial transmembrane potential and cell viability.

## Figures and Tables

**Table 1:** Integrated areas of representative vibration bands computed for exposed and not exposed samples, whose limits of integration were 3340−3260 cm^−1^, 2980−2947 cm^−1^, 2945−2900 cm^−1^, 2865−2835 cm^−1^, 1750−1730 cm^−1^, 1730−1710 cm^−1^, 1660−1640 cm^−1^, 1640−1610 cm^−1^, 1250−1180 cm^−1^, and 1120−1010 cm^−1^, respectively. Each value reported was averaged over a number of 26 spectra acquired at successive exposures. The cells of the table without values (n.d.) correspond to no appreciable change between exposed and not exposed samples or to not detected vibration band in the relative spectrum. The exposure levels refers to a time exposure of 4 h. *P < , 0.05, **P < 0.005 and ***P < 0.001 significant differences in comparison to control cells.

Exposure levels (mT)	Amide A 3295 cm^−1^	^as^CH_3_ 2961 cm^−1^	^as^CH_2_ 2925 cm^−1^	^s^CH_2_ 2853 cm^−1^	1740 cm^−1^	1725 cm^−1^	*β*-Sheet —1635 cm^−1^	*α*-Helix —1652 cm^−1^	^as^PO_2_^−^ 1235 cm^−1^	^s^PO_2_^−^ 1080 cm^−1^
0.00	1.00 ± 0.1	1.00 ± 0.07	1.00 ± 0.1	1.00 ± 0.1	1.00 ± 0.08	1.00 ± 0.1	1.00 ± 0.09	1.00 ± 0.1	1.00 ± 0.07	1.00 ± 0.07
0.050	0.67 ± 0.05	1.15 ± 0.06	1.26 ± 0.04	1.76 ± 0.03	1.55 ± 0.10	0.57 ± 0.05	n.d.	n.d.	n.d.	n.d.
0.81	0.32 ± 0.04	1.54 ± 0.15	1.84 ± 0.07***	2.02 ± 0.08***	2.03 ± 0.15*	1.25 ± 0.10	1.29 ± 0.05	1.13 ± 0.04	0.78 ± 0.07*	0.64 ± 0.06*
1.41	0.64 ± 0.04	1.67 ± 0.20***	2.69 ± 0.15***	3.08 ± 0.15***	2.88 ± 0.07	1.14 ± 0.04	1.95 ± 0.10	1.31 ± 0.08	n.d.	n.d.

**Table 2:** Mitochondrial transmembrane potential (exposed/control) ΔΨm and MTT test for cell viability, relative to three different magnetic flux density values after 4 h of exposure. *P < 0.05 and **P < 0.01 significant differences in comparison to control cells.

Exposure levels	0.00 mT	0.63 mT	0.81 mT	1.41 mT
ΔΨm (exposed/control)	1.00 ± 0.06	0.91 ± 0.06	0.82 ± 0.06	0.53 ± 0.05**
MTT (exposed/control)	1.00 ± 0.03	0.87 ± 0.03*	0.87 ± 0.02**	0.85 ± 0.02**
